# Manipulating the Fourier spectra of stimuli comprising a two-frame kinematogram to study early visual motion-detecting mechanisms: Perception versus short latency ocular-following responses

**DOI:** 10.1167/jov.23.10.11

**Published:** 2023-09-19

**Authors:** Boris M. Sheliga, Edmond J. FitzGibbon

**Affiliations:** 1 Laboratory of Sensorimotor Research, National Eye Institute, National Institutes of Health, Bethesda, MD, USA

**Keywords:** visual motion, perception and eye movements, contrast normalization

## Abstract

Two-frame kinematograms have been extensively used to study motion perception in human vision. Measurements of the direction-discrimination performance limits (*D_max_*) have been the primary subject of such studies, whereas surprisingly little research has asked how the variability in the spatial frequency content of individual frames affects motion processing. Here, we used two-frame one-dimensional vertical pink noise kinematograms, in which images in both frames were bandpass filtered, with the central spatial frequency of the filter manipulated independently for each image. To avoid spatial aliasing, there was no actual leftward-rightward shift of the image: instead, the phases of all Fourier components of the second image were shifted by ±¼ wavelength with respect to those of the first. We recorded ocular-following responses (OFRs) and perceptual direction discrimination in human subjects. OFRs were in the direction of the Fourier components’ shift and showed a smooth decline in amplitude, well fit by Gaussian functions, as the difference between the central spatial frequencies of the first and second images increased. In sharp contrast, 100% correct perceptual direction-discrimination performance was observed when the difference between the central spatial frequencies of the first and second images was small, deteriorating rapidly to chance when increased further. Perceptual dependencies moved closer to the OFR ones when subjects were allowed to grade the strength of perceived motion. Response asymmetries common for perceptual judgments and the OFRs suggest that they rely on the same early visual processing mechanisms. The OFR data were quantitatively well described by a model which combined two factors: (1) an excitatory drive determined by a power law sum of stimulus Fourier components’ contributions, scaled by (2) a contrast normalization mechanism. Thus, in addition to traditional studies relying on perceptual reports, the OFRs represent a valuable behavioral tool for studying early motion processing on a fine scale.

## Introduction

Two-frame kinematograms have been extensively used to study motion perception in human vision. Measurements of the direction-discrimination performance limits ([*D*_max_]; maximal spatial displacement) have been the primary subject of such studies (Baker & Braddick, 1982; Baker & Braddick, 1985; Boulton & Baker, 1991; Chang & Julesz, 1983a; Chang & Julesz, 1983b; Cleary & Braddick, 1990a; Cleary & Braddick, 1990b; Eagle & Rogers, 1996; Eagle & Rogers, 1997; Glennerster, 1998; Morgan, 1992; Morgan, Perry, & Fahle, 1997; Nakayama & Silverman, 1984; Todd & Norman, 1995; Tripathy, Shafiullah, & Cox, 2012). Considerably fewer studies, however, have asked how an incongruency in the spatial frequency (SF) content of individual frames affects visual motion processing (Bex & Dakin, 2002; Bex & Dakin, 2003; Hess, Bex, Fredericksen, & Brady, 1998; Ledgeway, 1996; Morgan & Mather, 1994). Of those, the paper by Ledgeway (1996) is the most systematic one.

Ledgeway (1996) asked the question: “How similar must the Fourier spectra of the frames of a random-dot kinematogram be to support motion perception?” A two-frame movie—random dot kinematogram (RDK)—was shown to the subjects in which an image shifted horizontally: left or right. Each frame was derived from the same RDK sample by bandpass filtering it with an isotropic 1-octave (log)Gaussian filter. For each frame, the filter had one of 12 different central SFs, from 0.75 to 9 cpd. All combinations (central SF and presentation order) of the filtered pairs of frames were randomly interleaved in a single block of trials. The horizontal step size of 1.875 arcmin between frames insured that whenever the two frames of any filtered RDK contained common SFs, these were always displaced by less than one half the cycle of their spatial periods to prevent any possible confounding effects of aliasing. The subjects reported the direction of perceived motion by a button press. The subjects showed nearly perfect direction discrimination as long as the difference between the central SFs of the two frames was within ±0.5 octave, followed by a sharp drop all the way toward chance level as the difference between the central SFs of the frames was increased further.

At first, we were puzzled by this pattern of results: stimuli used in the Ledgeway (1996) study had smooth (log)Gaussian shaped SF profiles, whereas the direction discrimination results had a strong binary flavor—“yes” or “no”—very much like implemented perceptual responses. But, on second thought, such discrepancy might have been expected: with suprathreshold stimuli, small central SF differences of images in the two frames might not have caused the psychometric function of percent correct judgments to start its descent toward the chance level. The motion stimulus was still above threshold and resulted in 100% correct discrimination, despite some deterioration of the motion percept quality. It is only when the difference between the central SFs was increased further, that the percent correct judgments started to collapse. To summarize, with suprathreshold stimuli, binary perceptual judgments might not be the right tool for uncovering the real shape of motion signal attenuation in the brain. We thought that short-latency ocular following responses (OFRs; Gellman, Carl, & Miles, 1990; Miles, Kawano, & Optican, 1986) could be quite handy in this situation. Studies utilizing OFRs made significant contributions in the visual motion research: these eye movements appear closely linked and provide a *quantitative* behavioral signature of neuronal activity at early stages of visual processing (for review, see Masson & Perrinet, 2012; Miles, 1998; Miles & Sheliga, 2010).

This paper tries to build on Ledgeway's (1996) results using the OFRs as a behavioral response. With the OFRs, however, tiny translational stimulus displacements used by Ledgeway (1996; 1.875 arcmin) were not an option: the displacements have to be much larger to evoke reliable OFRs. Making displacements larger, however, would result in confounding effects of spatial aliasing, predominantly at higher SFs^1^, and we know from previous work that spatial aliasing affects OFRs (Sheliga, Quaia, FitzGibbon, & Cumming, 2016). We thus adapted stimuli used by Quaia, Optican, & Cumming (2017) who successfully evoked OFRs by shifting the phases of all individual Fourier components of noise stimuli by a fixed angle. This approach is alias-free: a unidirectional phase shift (+90 degrees or −90 degrees; ¼ wavelength) was applied to phases of all Fourier components in the second frame. Experiments 1 and 2 report the OFRs and the perceptual direction discrimination responses using identical stimuli. Experiments 3 to 7 use the OFRs and test a wide range of stimulus central SFs and contrasts. We then propose a model that accounts for the OFR results.

Preliminary results of this study were presented in abstract form elsewhere (Sheliga, Quaia, FitzGibbon, & Cumming, 2022a).

## Materials and methods

Many of the techniques were similar to those used in this laboratory in the past (e.g. Sheliga, Chen, FitzGibbon, & Miles, 2005). Experimental protocols were approved by the institutional review board concerned with the use of human subjects. Our research was carried out in accordance with the Code of Ethics of the World Medical Association (Declaration of Helsinki), and informed consent was obtained for experimentation with human subjects.

### Subjects

Three subjects took part in this study: two were authors (B.M.S. and E.J.F.) and the third was a paid volunteer (J.C.) naïve as to the purpose of the experiments. All subjects had normal or corrected-to-normal vision. Viewing was binocular.

### Eye-movement recording

The horizontal and vertical positions of the right eye were recorded (sampled at 1 KHz) with an electromagnetic induction technique (Robinson, 1963). A scleral search coil embedded in a silastin ring (Collewijn, Van Der Mark, & Jansen, 1975) was placed in the right eye with the subject under topical anesthesia, as described by Yang, FitzGibbon, & Miles (2003). At the beginning of each recording session, a coil calibration procedure was performed using fixation targets monocularly viewed by the right eye.

### Visual display and stimuli

Dichoptic stimuli were presented using a Wheatstone mirror stereoscope. In a darkened room, each eye saw a computer monitor (HP p1230 21-inch CRT) through a 45 degree mirror, creating a binocular image 521 mm straight ahead from the eyes’ corneal vertices, which was also the optical distance to the images on the two monitor screens. Thus, the stereoscope was set up for equal vergence and accommodation demand. Each monitor was driven by an independent PC (Dell Precision 490), but the outputs of each computer's video card (PC NVIDIA Quadro FX 5600) were frame-locked via NVIDIA Quadro G-Sync cards. The monitor screens were each 41.8 degrees wide and 32.0 degrees high, had 1024 × 768-pixel resolution (i.e. 23.4 pixels/degrees directly ahead of each eye), and the two were synchronously refreshed at a rate of 150 Hz. Each monitor was driven via an attenuator (Pelli, 1997) and a video signal splitter (Black Box Corp., AC085A-R2), allowing presentation of black/white images with an 11-bit equidistant grayscale resolution (mean luminance of 20.8 cd/m^2^). Visual stimuli were seen through an approximately∼22 degrees by 22 degrees (512 × 512 pixels) rectangular aperture centered directly ahead of the eyes. The stimuli seen by the two eyes were always the same: we were not sure if we would need binocular manipulations to understand these responses and so we opted to use the stereoscope at the outset of the project.

**Figure 1.**
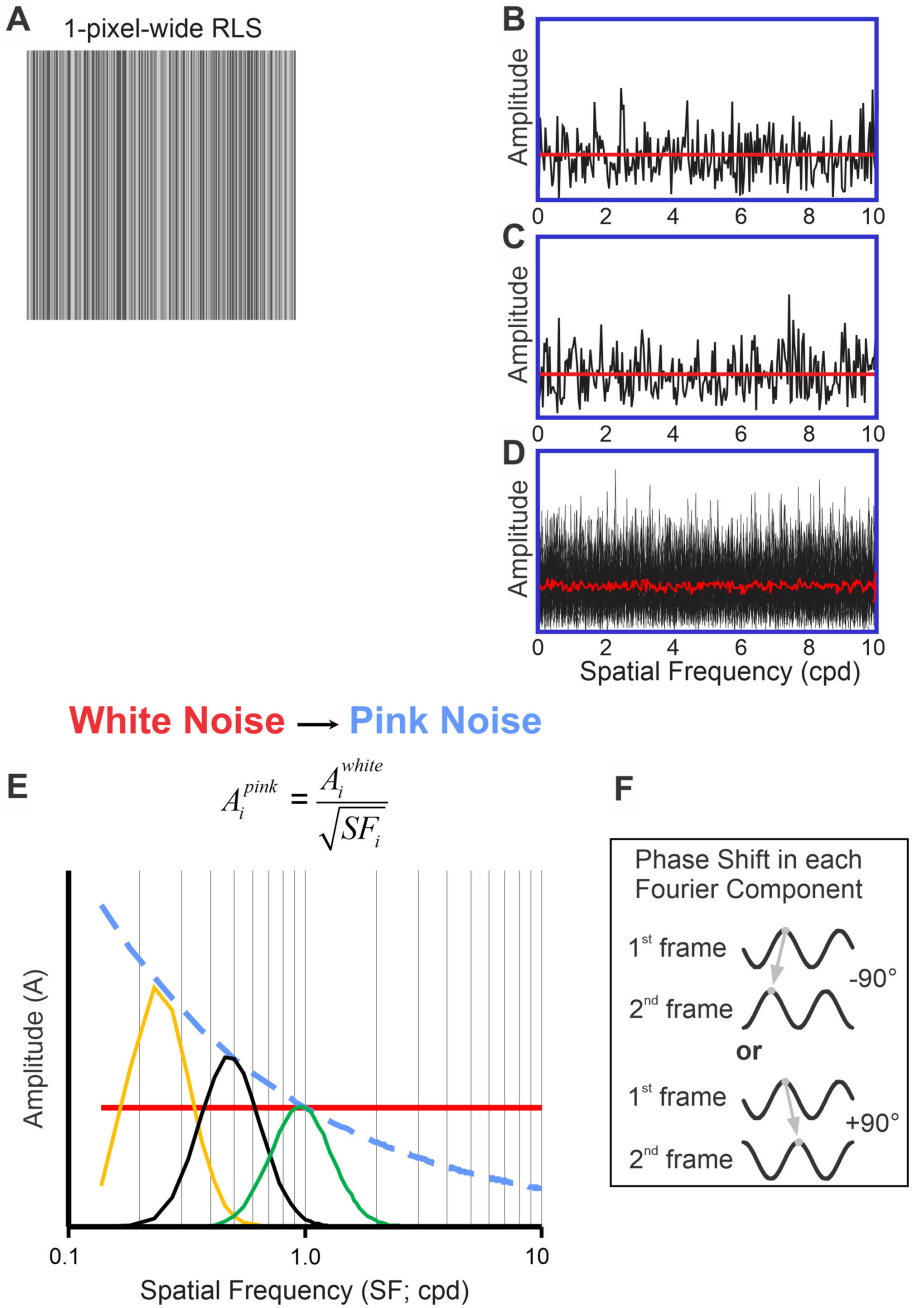
Noise stimuli. (**A**) An example of vertical one-dimensional white noise stimulus (random line stimulus [RLS]): a scaled version of a 22 degrees/22 degrees 1-pixel-wide pattern. (**B**) Noise sample.* Black:* Fourier composition; and *red:* the mean of the amplitudes of all the Fourier components. (**C**) Fourier composition of a different noise sample. (**D**) *Black:* Fourier composition of many random samples superimposed; and *red:* the mean amplitudes of different Fourier components across many random samples. (**E**) The dependence of a Fourier component's amplitude upon its SF. *Red solid line:* white noise sample; *blue dashed line:* pink noise sample; *orange, black, and green solid lines:* envelopes of 1-octave FWHM filters whose central SFs are 0.25, 0.5, and 1 cpd, respectively. (**F**) Phases of **all** Fourier components in the second frame of a pink noise sample are shifted leftward (−90 degrees) or rightward (+90 degrees) compared to those in the first frame.

The two-frame movies were visual stimuli presented to the subjects. Each frame contained a bandpass filtered vertical one-dimensional pink noise pattern (512 × 512 pixels). Creating a sample of such pattern involved a series of stimulus transformations, starting with a one-dimensional vertical binary white noise sample—Random Line Stimulus (RLS; Figure 1A)—constructed by randomly assigning a “black” or “white” value to each successive column of pixels. The actual luminance of black and white pixels was set to 0.25 and 0.75 of maximal luminance, respectively, resulting in 50% root mean square (RMS) contrast. RMS contrast was calculated using the following formula:

$$\begin{array}{c} RMS=\frac{\sqrt{\frac{\sum_{i=1}^{N}{\left(Lum_{i}-Lum_{mean}\right)}^{2}}{N}}}{Lum_{mean}}, \end{array}$$

using actual pixel luminance values (*Lum*). One-dimensional Fast Fourier Transformation (FFT) of such a sample along the horizontal axis—axis of motion—is shown in Figure 1B in black color. If one averages many random samples (shown in black in Figure 1D), the mean amplitudes of different Fourier components will be quite similar (shown in red in Figure 1D), hence a *white noise* stimulus. For a single sample, however, there is quite a lot of variability in the amplitudes of different Fourier components (black color, see Figure 1B), and those might be very different for another same-contrast random one-dimensional vertical RLS sample (Figure 1C). Such “undesirable” trial-to-trial variability in the amplitudes of Fourier components would necessarily result in the trial-to-trial variability in the magnitude of ocular responses to horizontal motion of these stimuli. We, therefore, calculated the mean of the amplitudes of all Fourier components (in a sample) and set this mean value as the amplitude of each Fourier component of this and all other randomly generated one-dimensional vertical RLS samples (shown in red in Figures 1B, 1C)^2^. Fourier components’ phases, on the other hand, were never altered and remained random. A white noise sample was then transformed into a pink noise one, by dividing the amplitude of each Fourier component by the square root of its SF. Consequently, as illustrated in Figure 1E, in pink noise samples (dashed blue line) Fourier components with SF <1 cpd had greater amplitudes, whereas those with SF >1 cpd had smaller amplitudes than the corresponding Fourier components in white noise samples (red solid line). The last step of stimulus transformation was applying an SF bandpass filter whose attenuation function was Gaussian on a log scale. Such a filter has central SF (cSF) and bandwidth, expressed as full width at half maximum (FWHM). In Figure 1E, three examples of 1-octave FWHM filters with 0.25, 0.5, and 1 cpd cSFs are shown by orange, black, and green traces, respectively. In different experiments, FWHM was set to either 1 or 2 octaves. Applying the filter of certain FWHM and cSF (cSF_1_) to a given pink noise sample would create the first frame of a two-frame movie. To create the second frame of the movie, the phases of **all** Fourier components in this same pink noise sample were shifted by 90 degrees in one direction (Figure 1F): leftward (−90 degrees; backward) or rightward (+90 degrees; forward). The filter was then applied, whose cSF (cSF_2_) was either lower or the same, or higher than cSF_1_, whereas the FWHM was the same. We used bandpass filtered vertical one-dimensional *pink noise* patterns, because, for a given FWHM of the filter, such patterns had very similar RMS contrast, regardless of the filter cSF: approximately 9.3% for 1-octave FWHM and approximately 13.3% for two-octave FWHM.

#### Experiment 1

Horizontal OFRs to a two-frame movie were measured. The cSF_1_ was 0.25 or 0.5 cpd. The cSF_2_ was up to ±2 octaves away from the cSF_1_, in one-half octave increments. In separately run sessions, the FWHM of the (log)Gaussian filter was 1 or 2 octaves, except for one 2-octave FWHM stimulus which was shown in all sessions allowing to normalize the OFR amplitudes, recorded in separately run sessions, to this common condition. A block of trials in 2-octave FWHM sessions had 36 randomly interleaved stimuli: 2 cSF_1_, 9 cSF_1_/cSF_2_ separations, and two directions of Fourier components’ shift (90 degrees; leftward or rightward). Thirty-eight randomly interleaved stimuli made a single block of trials in 1-octave FWHM sessions: 36, as described above, plus leftward and rightward 90 degrees Fourier components’ shift in 2-octave FWHM stimulus, which served as a common condition for the OFR amplitude normalization.

#### Experiment 2

Perceptual leftward/rightward direction discrimination to a two-frame movie was measured. We asked the subjects to indicate the perceived direction of pattern motion by a button press: left or right. Eye movements were not recorded in this experiment. FWHM of the (log)Gaussian filter was 1 or 2 octaves. The cSF_1_ was 0.25 cdp for 1-octave FWHM and 0.5 cpd for 2-octave FWHM. The cSF_2_ was up to ±2 octaves away from cSF_1_, in one-half octave increments. A block of trials had 36 randomly interleaved stimuli: 2 cSF_1_, 9 cSF_1_/cSF_2_ separations, and two directions of Fourier components’ shift (90 degrees; leftward or rightward).

#### Experiment 2a

Perceptual leftward/rightward direction discrimination to a two-frame movie was measured. The visual stimuli were the same as in experiment 2, although the subjects were instructed to report not only the perceived direction (left or right) but also the strength of pattern motion. Subjects were told to press the same button (left or right) for up to three (subject B.M.S.) or five times (subjects E.J.F. and J.C.) to quantify the strength of perceived motion: 1 = weak; 3 to 5 = strong. Eye movements were not recorded in this experiment. One-octave and 2-octave FWHM stimuli were used in separately run sessions. Thus, a block of trials in a given session (1-octave and 2-octave FWHM) had 18 randomly interleaved stimuli: 9 cSF_1_/cSF_2_ separations and two directions of Fourier components’ shift (90 degrees; leftward or rightward).

For any given stimulus, the direction discrimination percent correct was calculated using the following equation:

(1) $$\begin{array}{ccc} & & \mathrm{Percent}\mathrm{Correct}\\ & & =\left[\frac{R_{C}-R_{IC}}{2*M*\left(N_{R}+N_{L}\right)}+0.5\right]*100\%\; \end{array}$$

*R_C_* = number of button responses consistent with the shift direction (left button for leftward shift and right button for rightward shift). *R_IC_* = number of button responses inconsistent with the shift direction (right button for leftward shift and left button for rightward shift). *M* = maximal number of button presses allowed for evaluation of motion direction and strength: once in experiments 2 and 3 (subject B.M.S.) or five times (subjects E.J.F. and J.C.) in experiment 2a. *N_R_* and *N_L_* = number of presentations of rightward and leftward motion stimuli, respectively.

For experiment 2, Equation 1 simplifies to:

(11) $$\begin{array}{c} \mathrm{Percent}\mathrm{Correct}=\frac{R_{C}}{N_{R}+N_{L}}*100\%,\; \end{array}$$

a familiar simple form, which clearly matches the definition of “percent correct.” We retain this axis label when showing the results of experiment 2a, because Equation 1 provides the appropriate scaling in Experiment 2a as well: >50% up to 100% if *R_C_* *>* *R_IC_*, 50% if *R_C_* *=* *R_IC_*, and <50% down to 0% if *R_C_* *<* *R_IC_*.

#### Experiment 3

Horizontal OFRs to a two-frame movie were measured. Experiment 3 tested an 11-fold range of cSF_1_. The FWHM of the (log)Gaussian filter was 1 octave. The cSF_1_ was 0.125, 0.177, 0.354, 0.707, 1, or 1.414 cpd for subject B.M.S. The 1.414-cpd cSF_1_ was not used with subject E.J.F., 1.414- and 1-cpd cSF_1_ were not used with subject J.C.^3^. The cSF_2_ was up to ±1 octaves away from the cSF_1_, in one-half octave increments. Sixty-two (subject B.M.S.), 52 (subject E.J.F.), or 42 (subject J.C.) randomly interleaved stimuli made a single block of trials: six (subject B.M.S.), five (subject E.J.F.), or four (subject J.C.) cSF_1_, 5 cSF_1_/cSF_2_ separations, and two directions of Fourier components’ shift (90 degrees; leftward or rightward), plus leftward and rightward 90 degrees Fourier components’ shift in the stimulus from experiment 1 which served as the common condition for the OFR amplitude normalization.

#### Experiment 4

Horizontal OFRs to a two-frame movie were measured. Each frame contained a one-dimensional vertical sine wave grating of the same SF and contrast, although the phases of the gratings in the first and second frames differed by 90 degrees (¼-wavelength). SFs ranged from 0.625 to 1 cpd (from 0.625 to 0.5 cpd in subject J.C.) in octave increments. The RMS contrast was 2.8%, 5.6%, 11.3%, or 22.6% (4%, 8%, 16%, or 32% Michelson contrast, respectively). A block of trials had 42 (subjects B.M.S. and E.J.F.) or 34 (subject J.C.) randomly interleaved stimuli: five (B.M.S. and E.J.F.) or four (J.C.) sine wave SFs, four RMS contrasts, and two signs of the between-frame phase shift (±90 degrees), plus leftward and rightward 90 degrees Fourier components’ shift in the stimulus from experiment 1, which served as common conditions for the OFR amplitude normalization.

#### Experiments 5 to 7

Horizontal OFRs to a two-frame movie were measured. Experiments 5 to 7 tested a four-fold range of stimulus contrasts. The FWHM of the (log)Gaussian filter was 1 or 2 octaves. The cSF_1_ was 0.125 or 0.5 cpd. The cSF_2_ was ±1 octaves away from the cSF_1_, in one-half octave increments. A single block of trials had 42 randomly interleaved stimuli: two FWHMs, 2 cSF_1_, and 5 cSF_1_/cSF_2_ separations, two directions of Fourier components’ shift (90 degrees; leftward or rightward), plus leftward and rightward 90 degrees Fourier components’ shift in the stimulus from experiment 1, which served as the common condition for the OFR amplitude normalization. The amplitude of all Fourier components in randomly generated one-dimensional vertical binary RLS samples for experiments 5, 6, and 7 was, respectively, ½, ¾, and 1.5 times that used in experiments 1 to 3. So, the bandpass filtered vertical one-dimensional pink noise patterns’ RMS contrasts were approximately 4.7% or approximately 6.6% (1- or 2-octave FWHM, respectively), approximately 7.0% or approximately ∼9.9%, and approximately ∼13.8% or approximately 19.5% in experiments 5, 6, and 7, respectively.

### Procedures

Experimental paradigms were controlled by three PCs, which communicated via Ethernet (TCP/IP protocol). The first PC utilized a Real-time EXperimentation software (REX; Hays, Richmond, & Optican, 1982), which provided the overall control of the experimental protocol, acquisition, display, and storage of the eye-movement and manual (button pressing) data. Two other PCs utilized the Psychophysics Toolbox extensions of Matlab (Brainard, 1997; Pelli, 1997) and generated the visual stimuli.

**Figure 2.**
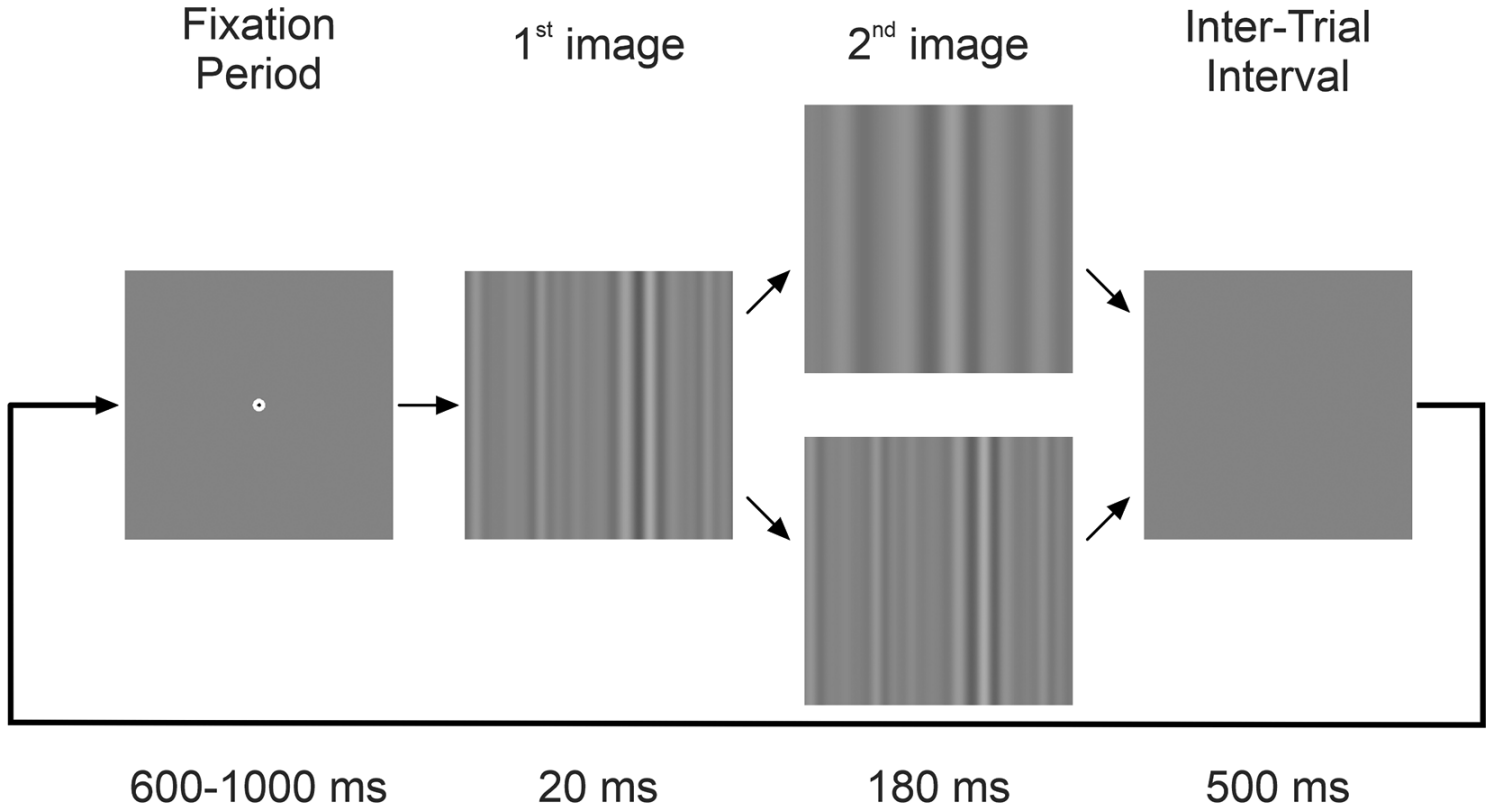
The temporal sequence of events in an experimental trial. See text for details. Two examples: Central SFs of the first image and the lower second image are the same; central SF of the upper second image is lower than that of the first image.

The temporal sequence of events in an experimental trial is shown in Figure 2. At the start of each trial, a central fixation target (diameter 0.25 degrees) appeared at the center of the otherwise uniform gray (luminance, 20.8 cd/m^2^) screen. In the OFR experiments (experiments 1 and 3-7), the subject's eye had to be continuously positioned within 2 degrees of the fixation target for a randomized period of 600 to 1000 ms to proceed, whereas in the perception experiments (experiments 2 and 2a), in which the eye position was not monitored, the fixation target stayed on the screen for 600 to 1000 ms and the subjects were instructed to look at it. The fixation target was then replaced by the first image of a (randomly selected from a look-up table) two-frame movie which stayed on the screen for 20 ms (3 video frames) and was followed by the second image, which stayed on the screen for an additional 180 ms (27 video frames). The screen then turned to uniform gray (luminance, 20.8 cd/m^2^) marking the end of the trial and the start of an inter-trial interval. The subjects were asked to refrain from blinking or shifting fixation except during the inter-trial intervals but were given no instructions relating to the motion stimuli. In the OFR experiments, a new fixation target appeared after a 500 ms inter-trial interval, signaling a new trial. If no saccades were detected (using an eye velocity threshold of 18°/s) for the duration of the trial, then the data were stored; otherwise, the trial was aborted and repeated within the same block. Upon completion of each trial in the perception experiments, the subjects were required to press the left or right button—one time in experiment 2 and three to five times in experiment 2a—of a three-button panel to report the perceived direction of motion. Subjects would then press the central button to initiate a new trial. Data collection occurred over several sessions until each condition had been repeated an adequate number of times to permit good resolution of the responses (through averaging).

### Data analysis

The calibration procedure provided eye position data which were fitted with second-order polynomials and later used to linearize the horizontal eye position data recorded during the experiment. Eye-position signals were then smoothed with an acausal sixth-order Butterworth filter (3 dB at 30 Hz) and mean temporal profiles were computed for each stimulus condition. Trials with micro-saccadic intrusions (that had failed to reach the eye-velocity cutoff of 18 degrees/second used during the experiment) were deleted. We utilized “position difference measures” to minimize the impact of directional asymmetries and boost the signal-to-noise ratio: the mean horizontal eye position with each leftward motion stimulus (90 degrees leftward phase shift in Fourier components of the second frame of the 2-frame movie) was subtracted from the mean horizontal eye position with the corresponding rightward motion stimulus (90 degrees rightward phase shift in Fourier components of the second frame of the 2-frame movie). As rightward eye movements were positive in our sign convention, OFRs in the direction of stimulus motion result in positive pooled measures. Mean eye velocity was estimated by subtracting position difference measures 10 ms apart (central difference method) and evaluated every millisecond. Response latency was estimated by determining the time elapsed since the appearance of the second frame of the two-frame movie to when the mean eye velocity first exceeded 0.1 degrees/second. The initial OFRs to a given stimulus were quantified by measuring the changes in the mean horizontal eye position signals—“OFR amplitude”—over the initial open-loop period, that is, over the period up to twice the minimum response latency. This window always commenced at the same time after the appearance of the second frame of the two-frame movie (“stimulus-locked measures”) and, for a given subject, was the same in all experiments reported in this paper: 64 to 128, 68 to 136, and 60 to 120 ms for B.M.S., E.J.F., and J.C., respectively. Bootstrapping procedures were used for statistical evaluation of the data and to construct 68% confidence intervals of the mean in the figures (these intervals were smaller than the symbol size in many cases and, therefore, not visible on many graphs).

## Results

### Experiment 1: OFRs

**Figure 3.**
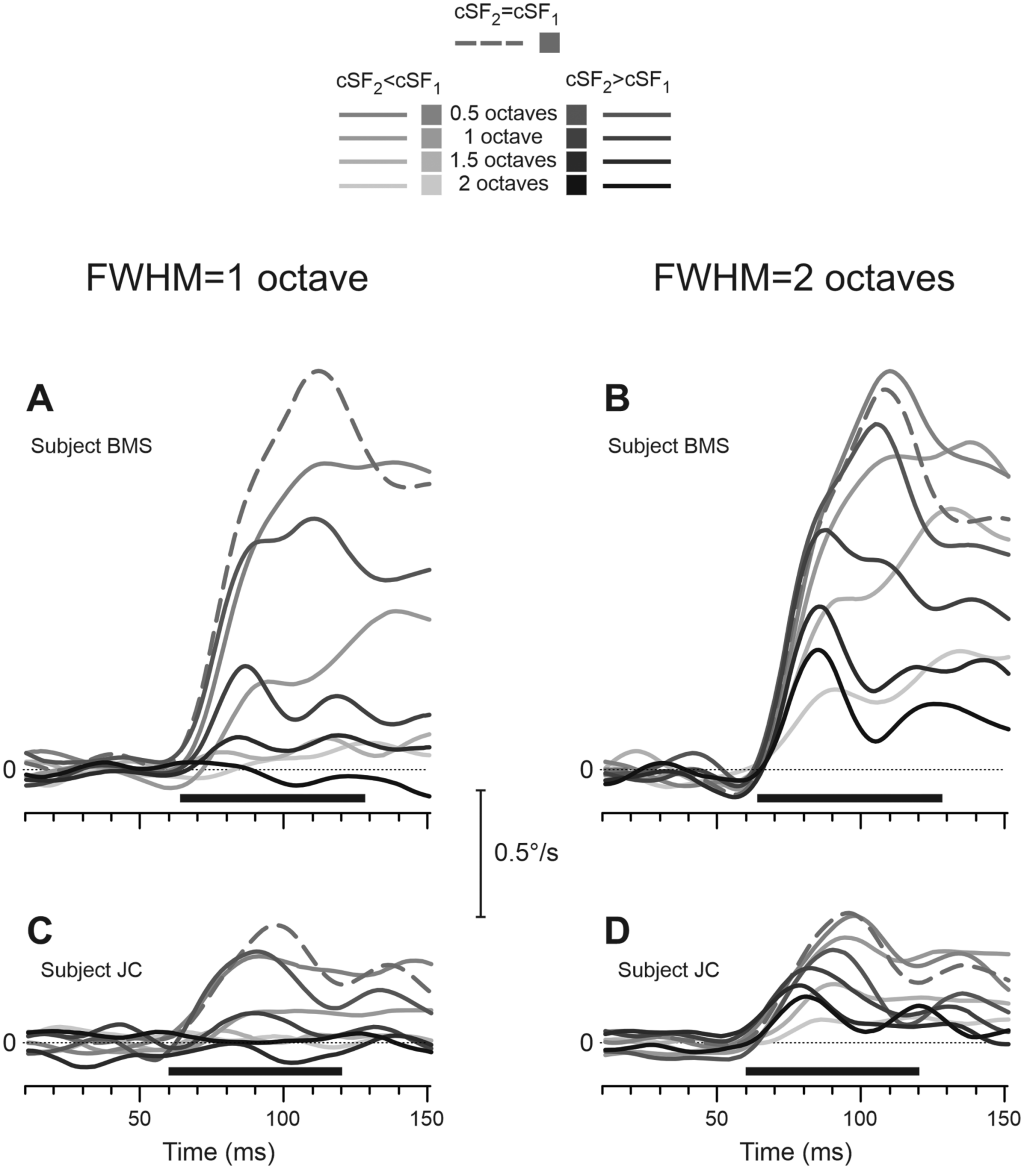
Experiment 1. Mean eye velocity profiles over time to 1-octave FWHM (**A**, **C**) and 2-octave FWHM (**B**, **D**) (log)Gaussian-filtered pink noise two-frame stimuli. The grayscale coding of velocity traces (see the insert) reflects the (log)difference between the central SFs of stimuli presented in the first and second frames: dashed medium-gray traces depict the OFRs in the cSF_2_ = cSF_1_ = 0.25 cpd condition. Progressively lighter-gray solid traces show conditions in which cSF_2_ was lower than cSF_1_ (cSF_2_ < cSF_1_) as the difference between them increased in one-half octave increments from 0.5 to 2 octaves. Progressively darker-gray solid traces depict conditions in which cSF_2_ was higher than cSF_1_ (cSF_2_ > cSF_1_) as the difference between them increased in one-half octave increments from 0.5 to 2 octaves. Each trace is the mean response to 77 to 90 (subject B.M.S.) and 75 to 112 (subject J.C.) repetitions of the stimulus. Abscissa shows the time from the appearance of the second frame; the *horizontal dotted line* represents zero velocity; the *horizontal thick black line* beneath the traces indicates the response measurement window.

Figure 3 shows mean horizontal OFR velocity profiles of two subjects—one of the authors (B.M.S.; see Figures 3A, 3B in the upper row) and the naïve subject (J.C.; see Figures 3C, 3D in the lower row)—to two-frame movies in which the (log)difference between cSF_1_ and cSF_2_ was systematically manipulated (noted by grayscale coding of velocity traces). Dashed medium-gray traces depict the OFRs in the cSF_2_ = cSF_1_ = 0.25 cpd condition. Progressively lighter-gray solid traces show conditions in which cSF_2_ was lower than cSF_1_ (cSF_2_ < cSF_1_) as the difference between them increased in one-half octave increments from 0.5 to 2 octaves. Progressively darker-gray solid traces depict conditions in which cSF_2_ was higher than cSF_1_ (cSF_2_ > cSF_1_) as the difference between them increased in one-half octave increments from 0.5 to 2 octaves. The left column of panels displays 1-octave FWHM data, and the right column displays 2-octave data. Although the OFR amplitudes of subject B.M.S. are approximately three times larger than those of subject J.C., the overall pattern of results in the two subjects is very similar. In 1-octave FWHM data, the OFRs plummet as soon as the (log)difference between cSF_1_ and cSF_2_ exceeds 0.5 octave (see Figures 3A, 3C), whereas for 2-octave FWHM data a decline in the OFR amplitude is much more gradual as the (log)difference between cSF_1_ and cSF_2_ is being increased (see Figures 3B, 3D). For 1-octave FWHM data, the cSF_1_ = cSF_2_ condition produced the strongest OFRs (dashed traces). For 2-octave FWHM data, however, the 0.5-octave cSF_2_ < cSF_1_ condition was either as effective as the cSF_1_ = cSF_2_ (subject J.C.) or the most effective one (subject B.M.S.). In general, in the difference-matched conditions, the OFRs were stronger for cSF_2_ < cSF_1_ than for cSF_2_ > cSF_1_, as evident from Figures 3B, 3D with lighter-gray traces rising above darker-gray ones.

**Figure 4.**
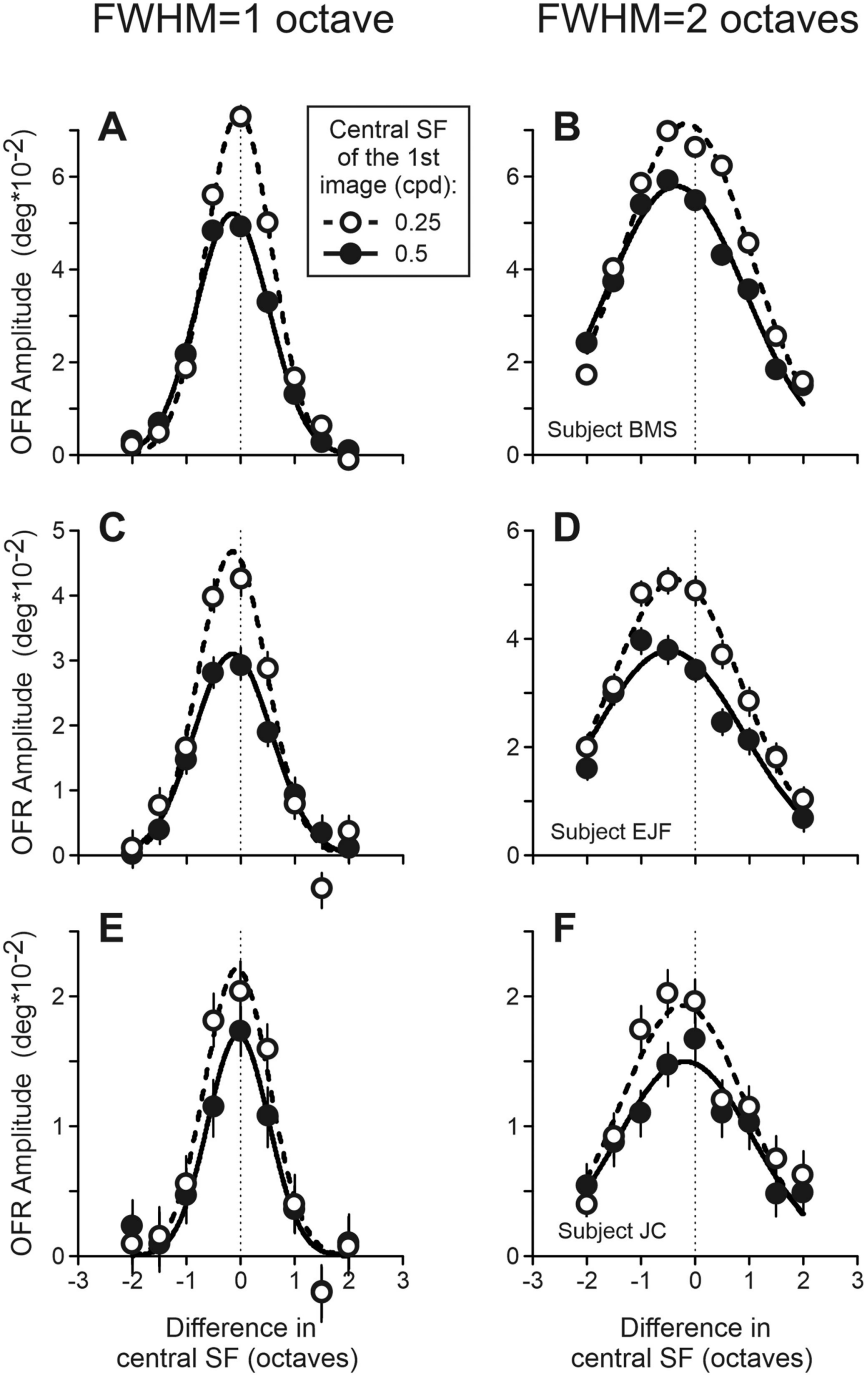
Experiment 1. Dependence of mean OFR amplitude on the (log)difference between the central SFs of stimuli presented in the first and second frames of a two-frame movie. The 0.25-cpd cSF_1_ data and fits: *open circles* and *dashed lines*; and 0.5-cpd cSF_1_ data and fits: *filled circles* and *solid lines*. The *left column* of panels: 1-octave FWHM data, and the *right column* of panels – 2-octave data. The *thin dashed vertical lines*: cSF_1_ = cSF_2_. Subject B.M.S. (**A, B;** 75–90 trials per condition; 68% confidence interval range = 0.0031 degrees–0.0046 degrees); subject E.J.F. (**C, D;** 81-119 trials per condition; 0.0041 degrees–0.0055 degrees); subject J.C. (**E, F;** 75-112 trials per condition; 0.0029 degrees–0.0047 degrees).

These features are quantified in six panels of Figure 4, which show OFR amplitude (ordinate and degrees) as a function of the (log)difference between the cSFs of stimuli presented in the first and second frames of a two-frame movie (abscissa and octaves). Negative/positive values on abscissa correspond to conditions when the cSF_2_ is lower/higher than the cSF_1_. The left column of panels displays 1-octave FWHM data, and the right column displays 2-octave data. Data of each subject occupy a single row. All these relationships were well fit by Gaussian functions (median *r^2^* = 0.970; *r^2^* range = 0.864–0.996). The 0.25-cpd cSF_1_ data and fits are shown by open circles and dashed lines, whereas 0.5-cpd cSF_1_ data and fits are shown by filled circles and solid lines. Standard deviations of fits for 2-octave FWHM data were approximately two2 times bigger than for 1-octave FWHM data (median = 2.0, range = 1.9–2.2), reproducing the ratio of filters’ FWHMs. Not surprisingly, the OFRs were the strongest when the cSF_1_ and cSF_2_ were similar. The fits, however, often peaked at negative cSF difference values (cSF_2_ < cSF_1_), rather than when the cSF_1_ and cSF_2_ were the same: median = (−0.29), range = (−0.17) to (−0.49) for 2-octave FWHM data (Figures 4B, 4D, 4F); median = (−0.11), range = (−0.03) to (−0.15) for 1-octave FWHM data (Figures 4A, 4C, 4E).

### Experiment 2: Perceptual judgments

**Figure 5.**
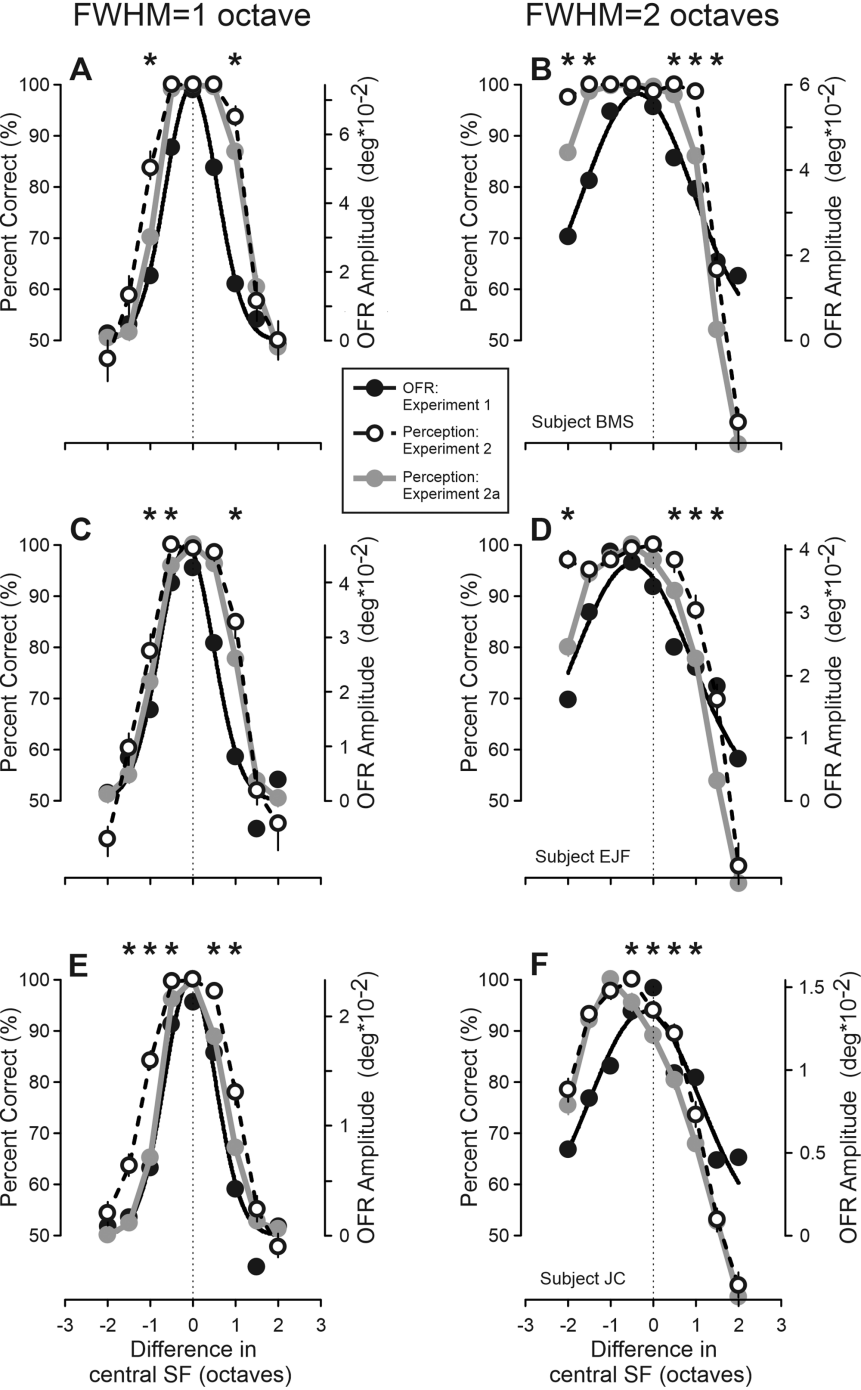
Experiments 2 and 2a. Dependence of the perceptual judgments’ percent correct on the (log)difference between the central SFs of stimuli presented in the first and second frames of a two-frame movie. Experiment 2: *open black circles* and *dashed lines*; experiment 2a: *filled grey circles* and *solid lines*. *Left column* of panels: 1-octave FWHM data, right column of panels – 2-octave data. *Thin dashed vertical lines*: cSF_1_ = cSF_2_. *Asterisks* on top of the graphs mark instances when a change in percent correct values between experiments 2 and 2a was statistically significant. Subject B.M.S. (**A, B;** 40, 61, and 60 trials per condition in experiment 2, and 1-octave and 2-octave FWHM stimuli in experiment 2a, respectively); subject E.J.F. (**C, D;** 66, 95, and 92 trials per condition); subject J.C. (**E, F;** 108, 75, and 107 trials per condition). In each panel, the OFR data, obtained for the same visual stimulation conditions, are replotted from Figure 4 for comparison: *filled black circles* and *solid lines*.

Open black circles in six panels of Figure 5 show changes in the direction discrimination perceptual judgments (left ordinate and percent correct) as a function of the cSF_1_/cSF_2_ difference (abscissa and octaves). As in Figure 4, negative/positive values on abscissa correspond to conditions when the cSF_2_ is lower/higher than the cSF_1_. For comparison, the OFR data for the same visual stimulation conditions are replotted from Figure 4 (filled black circles and solid lines; right ordinate and degrees). Unlike the OFRs, all subjects showed nearly perfect direction discrimination if cSF_1_/cSF_2_ differences were small, followed by a sharp drop toward the chance level when the cSF_1_/cSF_2_ difference was increased further. On the other hand, notable asymmetries found using 2-octave FWHM filters with the OFRs were replicated with perception (see Figures 5B, 5D, 5F). For 1-octave FWHM filters not much was evident either with OFRs or perception (see Figures 5A, 5C, 5E). Such pattern of results suggests that perceptual judgments rely, at least in part, on the same visual processing mechanisms as the short-latency OFRs.

### Experiment 2a: Perceptual judgments

The visual stimuli in this experiment were the same as in experiment 2, although the subjects were instructed to report not only the perceived direction but also the strength of pattern motion by pressing the same button (left or right) for up to three (subject B.M.S.) or five times (subjects E.J.F. and J.C.): 1 = weak and 3 to 5 = strong. The shapes of six cSF_1_/cSF_2_ difference dependencies in experiment 2a were quite similar to those observed in experiment 2, although the maximal percent correct rate was always less than 100%: from the 80.8% to 99.6% range. To assess quantitatively if the shapes of the dependencies in experiments 2 and 2a were different, we rescaled those of experiment 2a so that the maximal percent correct rate (separately for each dependence) now equaled 100%. For data of experiment 2, such rescaling was not necessary for three dependencies (maximal percent correct rate was 100%) and was minimal for the other three (maximal percent correct rates were 99.2%, 97.7%, and 97.2%). As can be seen in Figure 5, when so adjusted, experiment 2a dependencies (grey filled circles and solid lines) departed from high percent correct values sooner than those of experiment 2 (black open circles and dashed lines). Asterisks on top of the graphs mark instances when differences in experiments 2 and 2a percent correct values were statistically significant (bootstrapping). Thus, when the subjects were given an opportunity to quantify the strength of their motion percept, the shapes of the perceptual dependences became more similar to the OFR ones (see Figure 5, black filled circles and solid lines).

In one condition of both experiments 2 and 2a—2-octave FWHM filters and 2-cpd cSF_2_—the percent correct responses fell well below the chance level in all three subjects: the right-most data points in Figures 5B, 5D, and 5F. All subjects systematically perceived motion in the direction opposite to that of the actual phase shift of Fourier components. In addition, we cannot suggest a good explanation for this result. There is no reversal in the direction of the OFRs in this condition, so perceptual data show something clearly different. However, this condition is the only one, in which the second frame of a two-frame movie is dominated by Fourier components of very high SF, which is not the case for all other stimuli of this study. It is, therefore, possible that at very high SFs higher-order motion mechanisms might also be in play and affect perceptual judgments, whereas short-latency OFRs are known to be driven by the first-order mechanisms (luminance modulation) and largely immune to higher-order ones (e.g. Hayashi, Miura, Tabata, & Kawano, 2008).

### Experiments 3 and 4: OFRs

**Figure 6.**
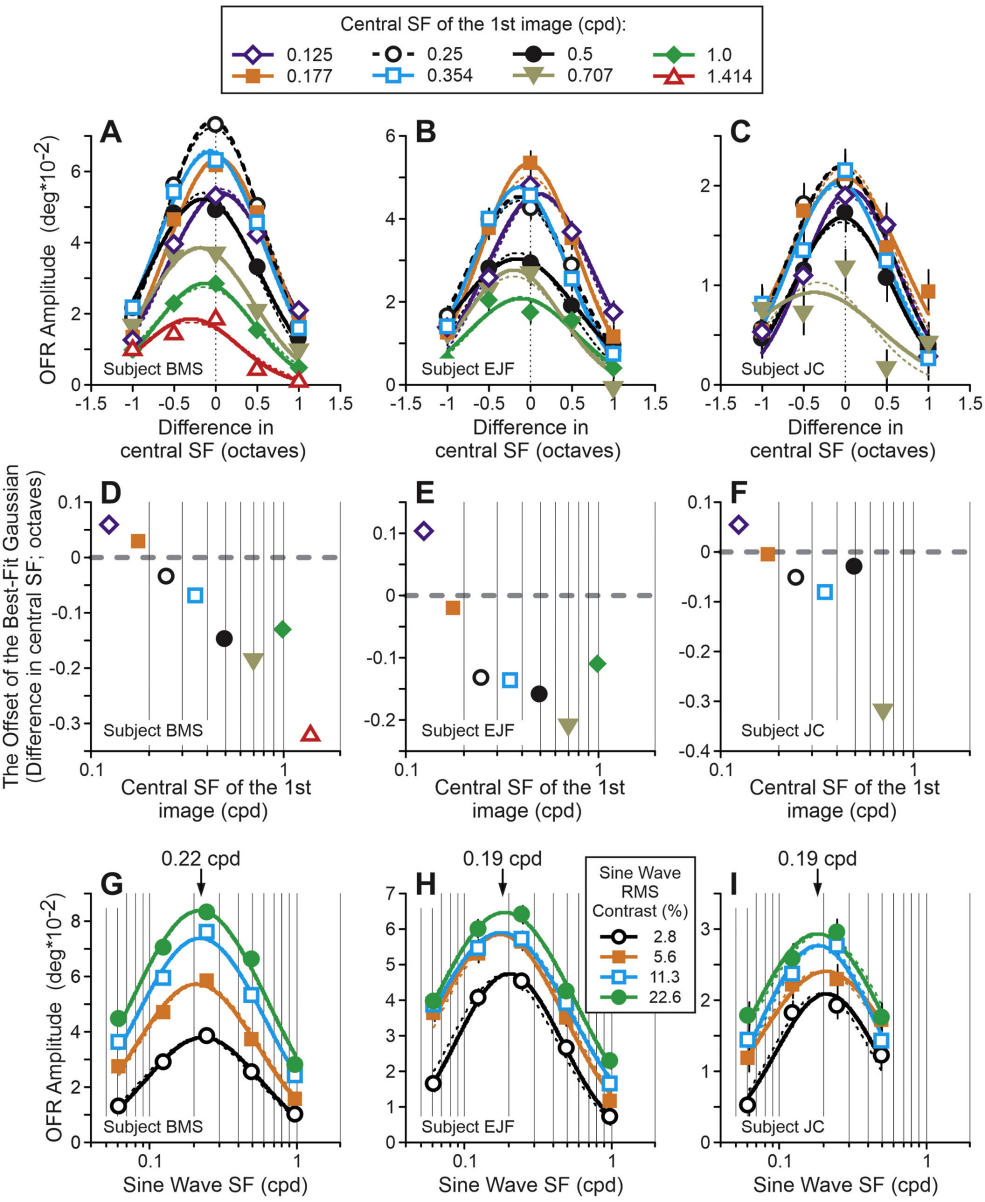
(**A–C**) Experiment 3. Dependence of mean OFR amplitude on the (log)difference between the central SFs of stimuli presented in the first and second frames of a two-frame movie. Different conditions of the central SF of the first image are symbol- and color-coded (see an upper rectangular insert). *Thin dashed vertical lines*: cSF_1_ = cSF_2_. Subject B.M.S. (**A;** 122–130 trials per condition; 68% confidence interval range = 0.0023 degrees to 0.0046 degrees); subject E.J.F. (**B;** 92–116 trials per condition = 0.0044 degrees to 0.0063 degrees); subject J.C. (**C;** 93–106 trials per condition; 0.0036 degrees to 0.0047 degrees). (**D–F**) Experiment 3. The offsets of the best-fit Gaussians as a function of the central SF of the image in the first frame of a two-frame movie. Symbols and colors as in (**A–C**). (**G–I**) Experiment 4. SF tuning for sine waves of different contrast (symbol- and color-coded; see a lower rectangular insert). Subject B.M.S. (**G;** 226–237 trials per condition; 68% confidence interval range = 0.0025 degrees to 0.0032 degrees); subject E.J.F. (**H;** 154–182 trials per condition; 0.0041 degrees to 0.0051 degrees); subject J.C. (**I;** 124–136 trials per condition; 0.0034 degrees to 0.0040 degrees).

In experiment 3 the cSF_1_ ranged from 0.125 to 1.414 cpd in one-half octave increments, the FWHM of the (log)Gaussian filter was always set to 1 octave, and the cSF_2_ was ±1 octave away from the cSF_1_ in one-half octave increments. So, this experiment tested an 11-fold range of cSF_1_. As do Figures 4 and 5, Figures 6A to 6C plot the OFR amplitude dependence upon the cSF_1_/cSF_2_ difference for three subjects. Different cSF_1_ conditions are symbol- and color-coded. The 0.25- and 0.5-cpd cSF_1_ data from experiment 1 are also included in the graphs. All these relationships were well fit by Gaussian functions (median *r^2^* = 0.986, *r^2^* range = 0.450–1.000; thick lines in Figures 6A–C). How similar were these fits? If one constrains the standard deviations of all Gaussian fits (for each subject) to be the same, changes are minimal: fits’ deterioration was not statistically significant (General Linear F-test: F(7, 16) = 1.08, F(6, 14) = 1.16, F(5, 12) = 0.35 for subjects B.M.S., E.J.F., and J.C., respectively); the fits are shown by thin dashed lines in Figures 6A to 6C. However, constraining the offsets of the best-fit Gaussians to be the same led to fit deterioration in two out of three subjects: F(14, 16) = 9.71 (*p* < 0.0001), F(6, 14) = 6.51 (*p* < 0.005), F(5, 12) = 0.91 (not significant [n.s.]) for subjects B.M.S., E.J.F., and J.C., respectively. Figures 6D to 6F plot the offsets of the best-fit Gaussians as a function of the cSF_1_. There was a clear dependence of the best-fit Gaussian offset upon the cSF_1_: for cSF_1_ higher/lower than approximately 0.2 cpd the offsets had negative/positive values, that is, Gaussian fits peaked in conditions when the cSF_2_ was lower/higher than the cSF_1_.

Of special note, is that this dependence crosses zero at around 0.2 cpd, that is, in the range of the peak of the OFR SF tuning curves for horizontally moving 1D vertical sine wave gratings of similar size (Sheliga, Quaia, FitzGibbon, & Cumming, 2020). Therefore, experiment 4 was designed to check if the OFR SF tuning curves were similar for a two-frame movie: each frame contained a one-dimensional vertical sine wave grating of the same SF and contrast, although the phases of the gratings in the first and second frames differed by 90 degrees (¼-wavelangth). Figures 6G to 6I show the results for three subjects. All SF dependences were very well fit by Gaussian functions (median *r^2^* = 0.986; *r^2^* range = 0.947–0.998; see thick lines in Figures 6G–6I; different contrast conditions are symbol- and color-coded). Constraining the offsets of Gaussian fits for sine waves of different contrasts to be the same, had minimal impact: the deterioration of the fits was not statistically significant (general linear F-test: F(3, 8) = 0.50, F(3, 8) = 3.86, F(3, 4) = 0.82 for subjects B.M.S., E.J.F., and J.C., respectively); these fits are shown by thin dashed lines in Figures 6G–6I. For subject B.M.S., the OFR SF tuning curve peaked at 0.22 cpd, for subjects E.J.F. and J.C. it was at slightly lower SF, 0.19 cpd. The dependence of the best-fit Gaussian offset upon the cSF_1_ (Figures 6D–6F) crosses zero at very similar values, and one can also see that the zero level is crossed at somewhat lower cSF_1_ for the latter two subjects.

### Experiments 5 to 7: OFRs

**Figure 7.**
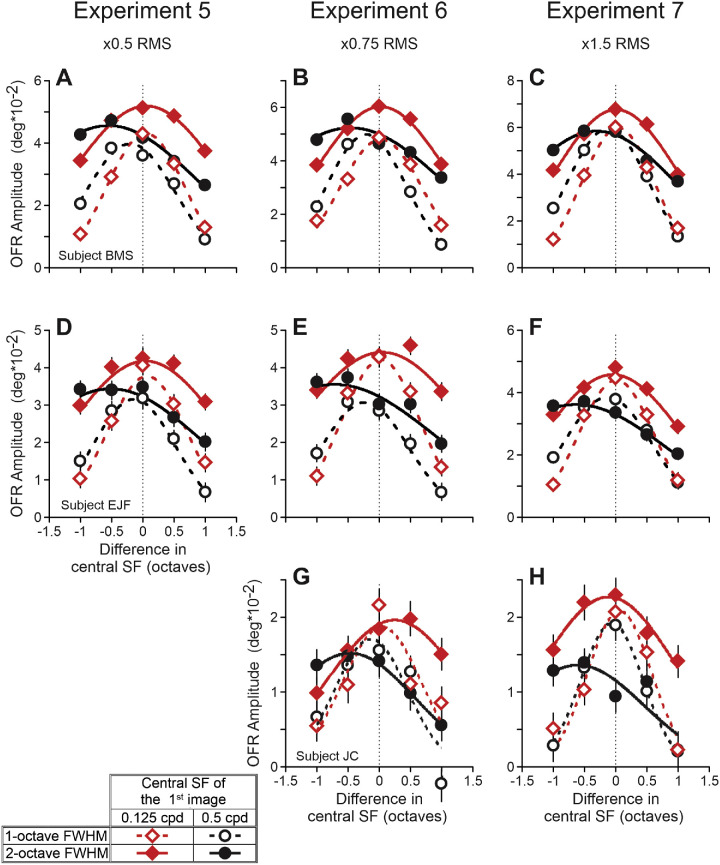
Experiments 5 to 7. Dependence of mean OFR amplitude on the (log)difference between the central SFs of stimuli presented in the first and second frames of a two-frame movie. Experiments 5 to 7 occupy panels of the *left*, *middle*, and *right* columns, respectively. The RMS contrasts of band-pass filtered vertical one-dimensional pink noise patterns for experiments 5, 6, and 7 were, respectively, ½, ¾, and 1.5 times of those used in experiments 1 to 3. The 0.125-cpd cSF_1_ data, Gaussian fits: *red diamonds*, *red lines*; 0.5-cpd cSF_1_ data, Gaussian fits: *black circles*, *black lines*. The 1-octave FWHM data: *open symbols*, *dashed lines*; 2-octave FWHM data: *filled symbols*, *solid lines*. See an insert. *Thin dashed vertical lines*: cSF_1_ = cSF_2_. Subject B.M.S. (**A–C;** 150–160 trials per condition; 68% confidence interval range = 0.0026 degrees to 0.0045 degrees); subject E.J.F. (**D–F;** 88–119 trials per condition; 0.0034 degrees to 0.0056 degrees); subject J.C. (**G, H;** 87–101 trials per condition; 0.0037 degrees to 0.0049 degrees).

Whereas experiment 3 tested an 11-fold range of cSF_1_, experiments 5 to 7 tested a four-fold range of stimulus contrasts: the RMS contrasts of band-pass filtered vertical one-dimensional pink noise patterns for experiments 5, 6, and 7 were, respectively, ½, ¾, and 1.5 times of those used in experiments 1 to 3, resulting in approximately 4.7 to 19.5% RMS contrast range (see Methods for details)^4^. Figure 7 is analogous to Figures 4 and 6A to 6C and shows changes in the OFR amplitude as a function of the cSF_1_/cSF_2_ difference. Data of each subject occupy a single row. Experiments 5 to 7 occupy panels of the left, middle, and right columns, respectively. All these relationships were well fit by Gaussian functions (median *r^2^* = 0.984, *r^2^* range = 0.750–0.999). The 0.125-cpd cSF_1_ data and fits are shown by red diamonds and red lines; 0.5-cpd cSF_1_ data and fits – by black circles and black lines. For 2-octave FWHM data, the symbols are filled, and the lines are solid; for 1-octave data – open symbols and dashed lines. Standard deviations of fits for 2-octave FWHM data were approximately two times bigger than for 1-octave FWHM data (median = 2.0, range = 1.6–2.3), reproducing the ratio of filters’ FWHMs. As evident from Figure 7, the OFR amplitude dependences on the cSF_1_/cSF_2_ difference are quite similar for experiments 5 to 7, although the magnitude of responses scaled with the contrast of stimuli used in these experiments (experiment 7 > experiment 6 > experiment 5). Pairwise comparisons—a total of seven comparisons; for example, experiment 5 versus experiment 6 for subject B.M.S.—using a single scaling free parameter, explained, on average, 94% of the variance (median *r^2^* = 0.936, *r^2^* range = 0.706–0.968). The OFRs were the strongest when the cSF_1_ and cSF_2_ were similar. However, for 0.125-cpd cSF_1_ data, the fits often peaked at positive cSF difference values (cSF_2_ > cSF_1_): median = (+0.04), range = (−0.12) to (+0.25). For 0.5-cpd cSF_1_ data, the fits always peaked at negative cSF difference values (cSF_2_ < cSF_1_): median = (−0.27), range = (−0.06) to (−0.72).

### Model

The data of experiments 1, and 3 to 7 were fit by the following equation:

(2) $$\begin{array}{ccc} & & \mathrm{OFR}=\frac{C^{k}}{C^{k}+C_{50}^{k}}\\ & & *\frac{{\left({\sum_{i=1}^{N}\left[OFR_{i}*{\left(\sqrt{C_{1,i}*C_{2,i}}\right)}^{m}\right]}^{n}\right)}^{\frac{1}{n}}}{\sqrt{\sum_{j=1}^{N}C_{1,j}^{m}*\sum_{j=1}^{N}C_{2,j}^{m}}}.\; \end{array}$$

*OFR_i_* is the response to a given Fourier component, derived from the Gaussian fit to the OFR SF tuning:

(21) $$\begin{array}{c} OFR_{i}=OFR_{MAX}*e^{-\frac{{\left[\log_{2}\left(SF_{i}\right)-\mu \right]}^{2}}{2*\sigma^{2}}},\; \end{array}$$

where *SF_i_* is a spatial frequency of this Fourier component, whereas *OFR_MAX_*, *μ,* and *σ* are the first three free parameters of the model. *OFR_i_* is multiplied by the geometric mean of contrasts of this component (*C_1,i_* and *C_2,i_*) in images of a two-frame movie. A single Fourier component contribution is raised to the power *m*, the fourth free parameter of the model. A power-law summation models a competition between contributions of different components, *n* being the fifth free parameter of the model. This is normalized by the geometric mean of the sums of the contributions of all Fourier components present in the first and second images (*C_1,j_* and *C_2,j_*), raised to the power *m*. *C* is the overall RMS contrast of the stimulus. *C_50_* and *k* are the last two—sixth and seventh— free parameters of the model. *N* is the number of Fourier components in the image (256 in our case).

For pure sine wave gratings, Equation 2 simplifies to:

(22) $$\begin{array}{c} OFR_{SW}=OFR_{SW(MAX)}*\frac{C^{k}}{C^{k}+C_{50}^{k}},\; \end{array}$$

which is a well-known Naka-Rushton equation (Naka & Rushton, 1966), successfully used to describe OFR contrast dependencies to pure sine wave stimuli in the past (Barthelemy, Perrinet, Castet, & Masson, 2008; Miura, et al., 2006; Quaia, FitzGibbon, Optican, & Cumming, 2018; Quaia et al., 2017; Quaia, Sheliga, Fitzgibbon, & Optican, 2012; Sheliga et al., 2005; Sheliga, Quaia, FitzGibbon, & Cumming, 2013). Thus, *C_50_* and *m* are the Naka-Rushton semi-saturation contrast and power term, respectively. *OFR_SW(MAX)_* is the maximal attainable response for sine wave of a given SF, calculated using Equation 2a.

For two-frame movies in which the cSF_1_ and cSF_2_ were the same, Equation 2 simplifies to:

(23) $$\begin{array}{c} \mathrm{OFR}=\frac{C^{k}}{C^{k}+C_{50}^{k}}*\frac{{\left({\sum_{i=1}^{N}\left[OFR_{i}*C_{i}^{m}\right]}^{n}\right)}^{\frac{1}{n}}}{\sum_{j=1}^{N}C_{j}^{m}},\; \end{array}$$

akin to a recently proposed model that successfully reproduced the disparity-vergence responses (DVRs) to broadband stimuli (Sheliga, Quaia, FitzGibbon, & Cumming, 2022b; see Discussion).

**Figure 8.**
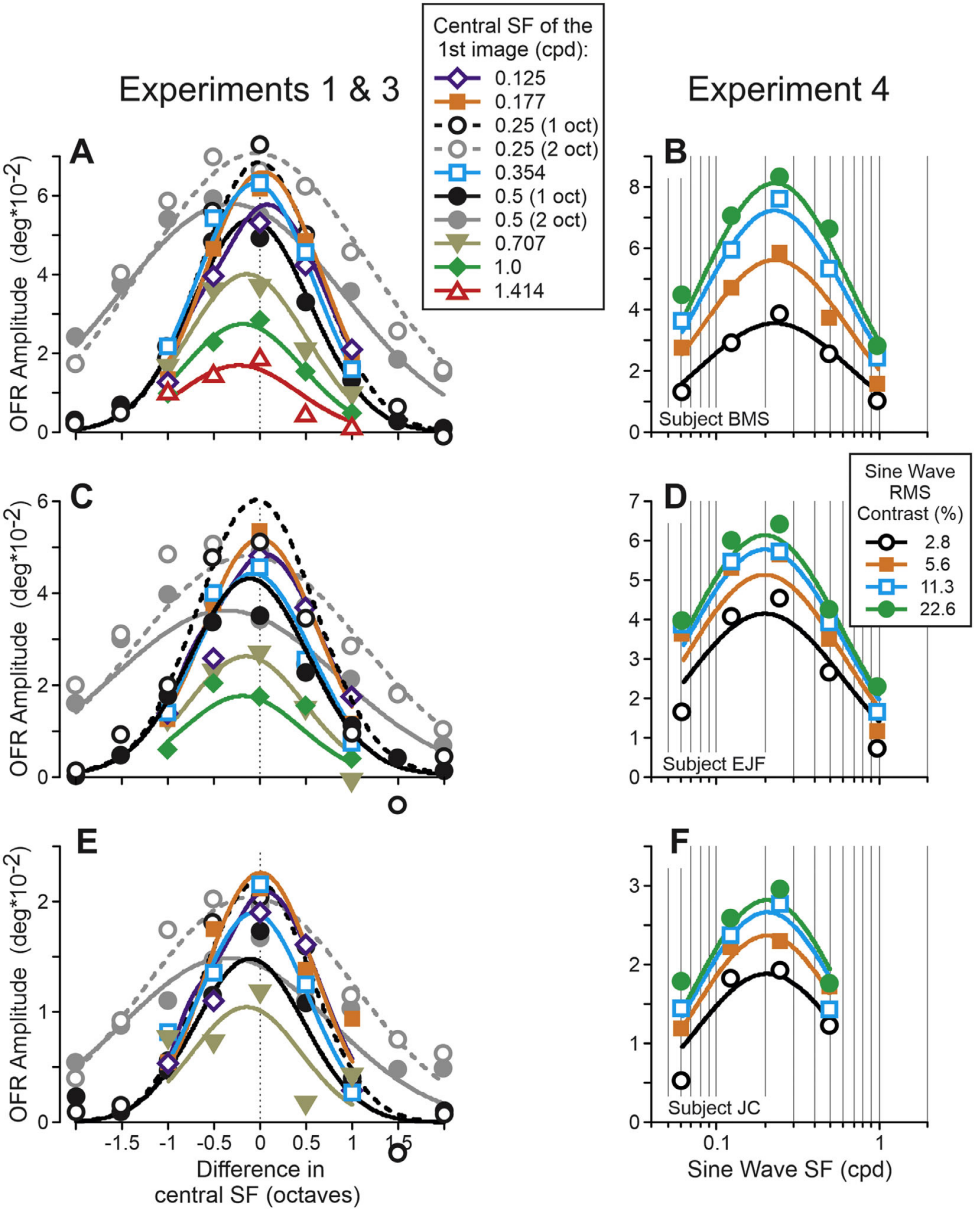
Experiments 1, 3, and 4. Equation 2 fits. Different experimental conditions are symbol- and color-coded (see rectangular inserts). *Thin dashed vertical lines*: cSF_1_ = cSF_2_.

**Figure 9.**
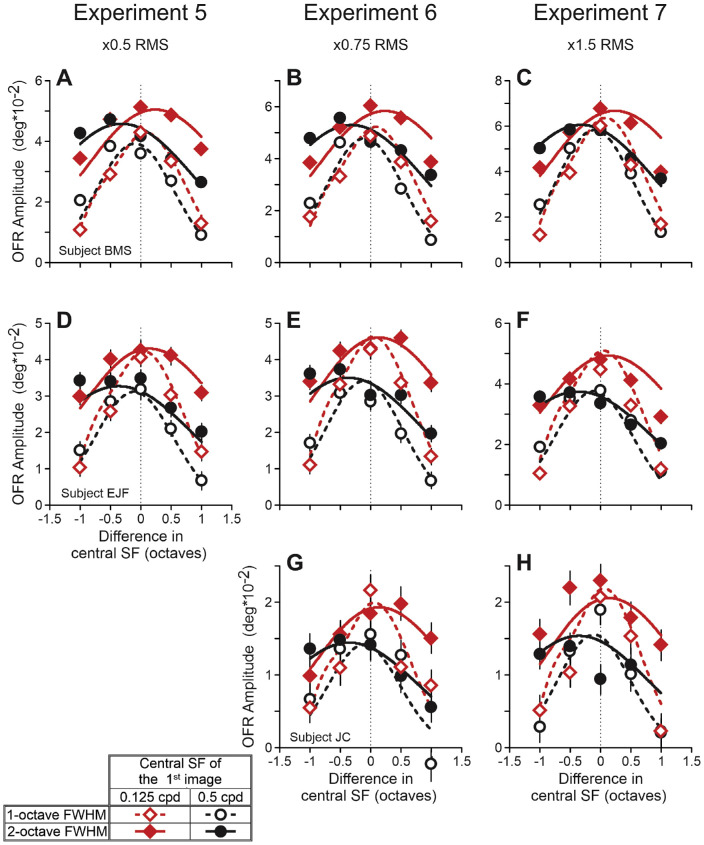
Experiments 5 to 7. Equation 2 fits. Different experimental conditions (as in Figure 7) are symbol- and color-coded (see an insert). Thin dashed vertical lines: cSF_1_ = cSF_2_.

Equation 2 provided good fits to the data – *r^2^* = 0.966, 0.930, and 0.897 for subjects B.M.S., E.J.F., and J.C., respectively. They are shown in Figure 8 for experiments 1, 3, and 4 and in Figure 9 for experiments 5 to 7. The Table 1 lists the best-fit values of free parameters: each subject's data in experiments 1, and 3 to 7 were fit by a single set of free parameters. Equation 2 has four free parameters beyond those that describe the linear stage – *m*, *n*, *C_50_*, and *k*. We used a general linear F-test to determine if these parameters were statistically justified, although *C_50_* and *k* were evaluated as a pair (it makes little sense to use a Naka-Rushton equation with only one free parameter). In one subject (B.M.S.) for one parameter (*n*) this was not significant (*p* = 0.25); the eight other cases were highly significant (*p* < 0.005 in all cases).

**Table 1.** Best-fit values of Equation 2 free parameters.

Subject	*OFR_max_* (°)	*µ (cpd)*	*σ (log2)*	*m*	*n*	*C_50_*	*k*	*r^2^*
**B.M.S.**	0.088	0.23	1.50	1.68	0.99	0.038	1.38	0.966
**E.J.F.**	0.065	0.20	1.59	1.70	1.05	0.017	1.09	0.930
**J.C.**	0.029	0.21	1.48	1.99	1.10	0.018	1.20	0.897

## Discussion

### Model

Equation 2 reproduced the OFRs to a wide range of broadband stimuli as well as pure sine waves. The model posits an operation of two factors: (1) an excitatory drive determined by a power law summation of contributions of stimulus Fourier components, scaled by (2) a contrast normalization mechanism. The inspection of the dependency of the OFR amplitude upon the cSF difference of stimuli presented in the first and second frames (see Figures 4, 6, 7) revealed that the best-fit (log)Gaussians often peaked at non-zero difference values, specifically when the cSF_2_ was closer to the value at which the OFRs to sine wave gratings were maximal (see Figures 6G–6I). These effects were stronger for 2-octave FWHM stimuli. All such observations provided a hint that the OFRs to broadband stimuli is determined by a summation of contributions of different Fourier components. Such summation is an integral part of Equation 2, which was very successful in reproducing the OFRs observed in our experiments. In Equation 2, the summation contains a nonlinearity modeled by the exponent *m*. In all subjects, the best-fit values of this free parameter were significantly higher than one (see the Table 1; *p* < 10^−14^), implying “winner-take-all” interactions, caused by differences in components’ contrasts. We reported such winner-take-all interactions in our earlier studies (Sheliga, Kodaka, FitzGibbon, & Miles, 2006; Sheliga et al., 2020). In several earlier studies, however, fits to the data were the best if the model entertained a weighted summation of the components, that is, when the contributions of Fourier components were weighted based on their SF: ∑OFR_i_*(W_i_*C_i_)^*m*^ instead of ∑$OFR_{i}*C_{i}^{m}$. In the Sheliga et al. (2022b) study, the best fits to the data were achieved when the weights of Fourier components were modeled by a power function of SF, adding one free parameter to the model (Equation 3b; Sheliga et al., 2022b). When we added such weights to Equation 2, the improvements in fits were not statistically significant for all subjects (*p* > 0.05; general linear F-test). In the Sheliga et al. (2020) study, the best fits to the data were achieved when *Wi = function(SF_i_)* was the product of two functions: an exponential and an inverted cumulative Gaussian function, adding three additional free parameters to the model (Equations 6 and 7; Sheliga et al., 2020). When we added such weight functions to Equation 2, the improvements in fits were statistically significant for subject E.J.F. (*p* < 0.01; F(3, 131) = 4.16), but not for the other two subjects (*p* = 0.18, F(3, 136) = 1.63 and *p* = 0.09, F(3, 102) = 2.23 for subjects B.M.S. and J.C., respectively). It is possible that the impact of Fourier component weights in the current study was minimal because we used narrow band-pass stimuli (1- and 2-octave FWHM), and the effects of components’ weights were masked by substantial differences in components’ contrasts. The Power law summation mechanism was successfully applied to account for spatial summation properties of neurons in cortical motion area MT (Britten & Heuer, 1999). It was also used previously to model the OFRs to the motion of white noise stimuli (Sheliga et al., 2016). Applying this mechanism in the current study significantly improved the fits of two subjects as well, although the actual values of free parameter *n* exceeded one (which corresponds to linear summation) not by much (1.05 and 1.10 for subjects E.J.F. and J.C., respectively). The contrast normalization probably reflects divisive inhibition among populations of cortical neurons sensitive to different SF components of visual stimuli (Britten & Heuer, 1999; Carandini & Heeger, 1994; Carandini, Heeger, & Movshon, 1997; Heeger, 1992; Heuer & Britten, 2002; Simoncelli & Heeger, 1998).

### Perception versus OFR

Using two different behavioral measures—eye movements (OFRs) and perceptual motion direction discrimination—this study utilized two-frame band-pass filtered visual stimuli to ask how an incongruency in the SF content of the individual frames affects visual motion processing. With the OFRs, as the difference between the central SFs of the first and second images was increased, a (log)Gaussian-shaped decline in amplitude was observed, whose standard deviation scaled with that of the filter. In sharp contrast, 100% correct perceptual direction-discrimination performance was observed when the cSF_1_/cSF_2_ difference was minor, deteriorating rapidly to chance when the difference was increased further. This perceptual outcome replicates observations of Ledgeway (1996), although, in our study, there was no actual leftward-rightward shift of the image: instead, the phases of all Fourier components of the second image were unidirectionally shifted with respect to those of the first. The discrepancy in the results obtained using eye movements and perception was, most likely, due to the fact that we utilized suprathreshold stimuli, and a slight deterioration of motion percept quality—caused by small central SF differences of images in the two frames—did not lead to a decline in percent correct judgments: the motion stimulus was still well above threshold and, hence, 100% correct motion direction discrimination was observed. This account of perception/eye movement results’ discrepancy is supported by the fact that allowing subjects to grade the saliency of perceived motion—that is, *quantifying* the judgment—did alter perceptual dependencies moving them closer to the OFR ones. Thus, in addition to studies relying on perceptual reports, the OFRs represent a valuable behavioral tool for studying early motion processing on a fine scale. Others, using a variety of behavioral contexts, observed diverging results when using and comparing the OFRs and perception (Glasser & Tadin, 2014; Simoncini, Perrinet, Montagnini, Mamassian, & Masson, 2012). This paper, on the other hand, provides an example of an experimental situation in which certain response properties—that is, response asymmetries—originating at early stages of visual processing (and thus reflected in the OFRs) are preserved in the hierarchy of visual processing stages and manifest themselves in perceptual judgments.

## Footnotes

1 A translational displacement of D_x_ degrees would result in a corresponding phase shift of *2π*f*D_x_* radians for a Fourier component, whose SF is *f.* Spatial aliasing results if such phase shift exceeds *π.*

2 A lookup table contained 250 randomly generated one-dimensional vertical binary RLS samples.

3 OFRs of subjects E.J.F. and J.C. were very small at these SFs.

4 As evident in Figures 3E, 3F, and 5C, the OFR amplitudes of subject J.C. in experiments 1 and 3 were quite small, so we opted to exclude this subject from participating in the experiment using the lowest contrast (experiment 5).
